# Supplementary material for: A Pilot Study on the Effects of Transcranial Direct Current Stimulation on Brain Rhythms and Entropy during Self-Paced Finger Movement using the Epoc Helmet
Source: Front Hum Neurosci. 2017 Apr 25;11:201. doi: 10.3389/fnhum.2017.00201 (PMC5408787; doi:10.3389/fnhum.2017.00201)
Supplement: Supplementary file 1 [file Data_Sheet_1.zip › Complementary_results/Explanatory file to CL-Ratio_average_PSD_windows.pdf]

Explanatory file to: *"CL-Ratio_average_PSD_windows"*

EEG signal processing:

- EEG .csv file is read by Matlab.
- Data points 1 to 23040 (180 seconds * 128Hz) are kept.
- Each channel is mean subtracted and multiplied by $0.5*10^{-6}$ (to convert values to Volts).
- Left-handed subjects get the left channels (AF3, F7, F3, FC5, T7, P7, O1) inverted with the right channels (AF4, F8, F4, FC6, T8, P8, O2).
- A forth order Butterworth band pass filter is applied with a bandwidth of 1 to 50Hz.
- Signal is split into 2 seconds time windows.
- Time windows are multiplied by a Hanning window.
- Every time windows DFT is computed using a FFT with a zero padding of 2^9.
- The PSD is acquired by taking the square of the absolute value of the DFT multiplied by 1/(sampling frequency * number of values).
- The PSD is averaged across all windows.
- From the average PSD, the Alpha (8-12Hz) and Beta (12-30Hz) frequency bands are computed using trapezoidal integration. Each band was divided by the complete integral from 1 to 45 Hz.
- From these bands, the Beta/Alpha ratio is computed. There is one ratio per channel, condition and subject.
- 4 new channels are created by averaging the ratio from 3 electrodes: AF3-F3-F7, AF4-F4-F8, F3-F7-FC5, F4-F8-FC6.
- The Laterality Coefficient (LC ratio) is computed using the following formula:

  $$LC = \frac{C - I}{C + I}$$

  Where *C* is Beta/Alpha ratio of the contralateral limb and *I* is the Beta/Alpha ratio of the ipsilateral limb.

Statistical analysis:

- Normality is assessed using a Shapiro-Wilk test on mean normalized and variance-standardized data.
- ANOVA on ranks is made using a Kruskal-Wallis test.
- Normal and equal variance data group are also tested using a one-way ANOVA.
- Multi-comparison testing is done using the Student-Newman-Keuls method.

Results:

- ANOVA on ranks showed no statistical difference between these groups and no effect of tDCS.
